# A flexible MHC class I multimer loading system for large-scale detection of antigen-specific T cells

**DOI:** 10.1084/jem.20180156

**Published:** 2018-05-07

**Authors:** Jolien J. Luimstra, Malgorzata A. Garstka, Marthe C.J. Roex, Anke Redeker, George M.C. Janssen, Peter A. van Veelen, Ramon Arens, J.H. Frederik Falkenburg, Jacques Neefjes, Huib Ovaa

**Affiliations:** 1 Oncode Institute and Department of Cell and Chemical Biology, Leiden University Medical Center, Leiden, Netherlands; 2 Core Research Lab, the Second Affiliated Hospital, School of Medicine, Xi'an Jiaotong University, Xi'an, China; 3 Department of Cell Biology II, Netherlands Cancer Institute, Amsterdam, Netherlands; 4 Department of Immunohematology and Blood Transfusion, Leiden University Medical Center, Leiden, Netherlands; 5 Center for Proteomics and Metabolomics, Leiden University Medical Center, Leiden, Netherlands; 6 Department of Hematology, Leiden University Medical Center, Leiden, Netherlands

## Abstract

Adaptive immunity is initiated by T cell recognition of specific antigens presented by major histocompatibility complexes (MHCs). MHC multimer technology has been developed for the detection, isolation, and characterization of T cells in infection, autoimmunity, and cancer. Here, we present a simple, fast, flexible, and efficient method to generate many different MHC class I (MHC I) multimers in parallel using temperature-mediated peptide exchange. We designed conditional peptides for HLA-A*02:01 and H-2K^b^ that form stable peptide–MHC I complexes at low temperatures, but dissociate when exposed to a defined elevated temperature. The resulting conditional MHC I complexes, either alone or prepared as ready-to-use multimers, can swiftly be loaded with peptides of choice without additional handling and within a short time frame. We demonstrate the ease and flexibility of this approach by monitoring the antiviral immune constitution in an allogeneic stem cell transplant recipient and by analyzing CD8^+^ T cell responses to viral epitopes in mice infected with lymphocytic choriomeningitis virus or cytomegalovirus.

## Abstract (precis)

Luimstra et al. describe a temperature-mediated peptide exchange method for generating many different epitope-specific MHC class I multimers in parallel. This simple and versatile technology allows fast and efficient production of MHC I reagents for immune monitoring of T cell responses.

## Graphical Abstract

**Figure:**
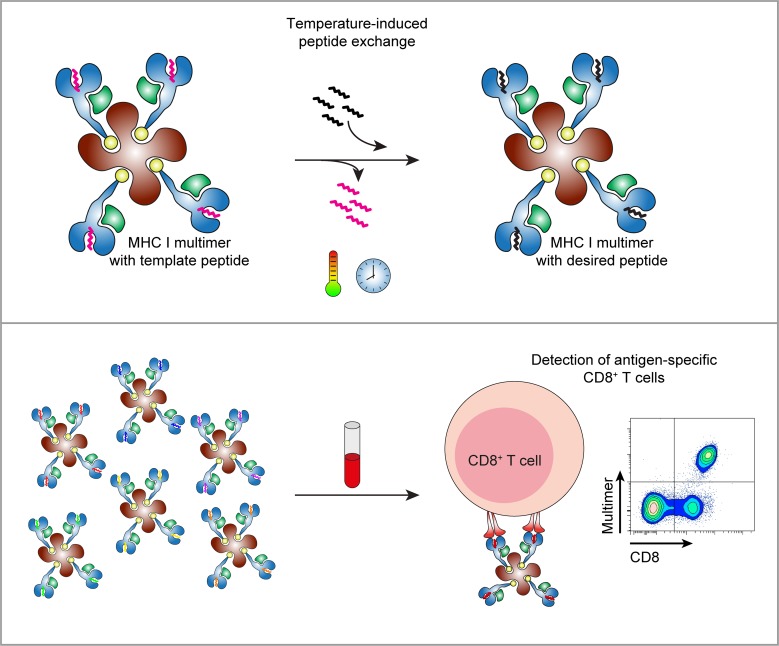

## Introduction

Immune surveillance is mediated by MHC class I (MHC I) complexes that bind intracellular peptides for presentation to CD8^+^ T lymphocytes. This ability to distinguish between self and foreign is fundamental to adaptive immunity, and failure can result in the development of autoimmune disease. During life, humans are under continuous attack by pathogens, such as viruses. Some of them establish lifelong infections, where the virus persists in a latent state without causing symptoms, but occasionally reactivates. One class of such viruses causing recurring infections is the herpesviruses (Grinde, 2013). Normally, reactivation does not lead to disease, because the infection is rapidly cleared by T cells upon recognition of viral antigens. However, in the context of transplantation, when patients are immunocompromised, reactivation of herpesviruses such as CMV or EBV can result in serious health threats (Broers et al., 2000; Green et al., 2016). It is therefore important to monitor virus-specific T cell numbers in transplant recipients to follow the fate of the recurring infections and to decide if intervention is needed.

Since their first use in 1996 by Altman et al., MHC multimers, oligomers of MHC monomers loaded with antigenic peptides and tagged with fluorochromes, have been the most extensively used reagents for the analysis and monitoring of antigen-specific T cells by flow cytometry (Altman et al., 1996). However, multimer generation involves many time-consuming steps, including expression of MHC I heavy chain and β2-microglubulin in bacteria, refolding with a desired peptide, purification, biotinylation, and multimerization (Altman et al., 1996). Initially, all these steps had to be undertaken for every individual peptide–MHC I complex, because empty MHC I molecules are unstable (Ljunggren et al., 1990). This prompted the search for ways to generate peptide-receptive MHC I molecules at will for the parallel production of multiple MHC I multimers from a single input peptide–MHC I complex. Several techniques aimed at peptide exchange on MHC I have been developed by us and by others, including dipeptides as catalysts or periodate or dithionite as chemical triggers to cleave conditional ligands in situ, after which peptide remnants can dissociate to be replaced by a peptide of choice (Rodenko et al., 2009; Amore et al., 2013; Choo et al., 2014; Saini et al., 2015). Alternatively, MHC I monomers are prepared with a photocleavable peptide that gets cleaved upon UV exposure, after which MHC I molecules can be loaded with peptides of choice and subsequently multimerized (Rodenko et al., 2006; Toebes et al., 2006; Bakker et al., 2008). This approach has facilitated the discovery of a myriad of epitopes and the monitoring of corresponding T cells (Toebes et al., 2006; Hadrup et al., 2009; Andersen et al., 2012; Bentzen et al., 2016). However, UV exchange technology requires the use of a photocleavable peptide and a UV source. UV exposure and ligand exchange are not compatible with fluorescently labeled multimers, and the biotinylated peptide-loaded MHC I molecules need to be multimerized on streptavidin after peptide exchange. Other disadvantages include the generation of reactive nitroso species upon UV-mediated cleavage and photodamage of MHC I and/or exchanged peptides, while the generated heat causes sample evaporation (Pattison et al., 2012).

To develop a faster, more convenient technology for peptide exchange on multimers, we explored our original observation that, early in MHC I assembly, low-affinity peptides continuously bind and dissociate from MHC molecules until a high-affinity/low–off-rate peptide is bound for presentation (Garstka et al., 2015). This process was strongly dependent on temperature: low-affinity peptides that stably associated at low temperature were released at slightly elevated temperatures and replaced with higher-affinity peptides (De Silva et al., 1999; Garstka et al., 2015). Here, we describe a direct application of this observation: the design of peptides with a low off-rate at 4°C that in a temperature-dependent manner can be exchanged for exogenous peptides of interest. We provide proof-of-concept for H-2K^b^ and HLA-A*02:01 multimers, representatives of dominant mouse and human MHC alleles, respectively. From a single standard batch of these MHC I multimers, we generated, within hours, multiple correctly loaded MHC I multimers, just by incubation with selected peptides at a defined temperature. We made many different MHC multimers to detect specific T cell responses in virus-infected mice and to measure T cell kinetics against various viral reactivations in a human transplant recipient. Temperature-exchangeable MHC I multimers will provide simple, fast, and convenient tools for epitope discovery and immune monitoring of large sets of potential antigenic peptides.

## Results

### Identification of peptide–MHC I combinations suitable for temperature exchange

**Figure 1.**
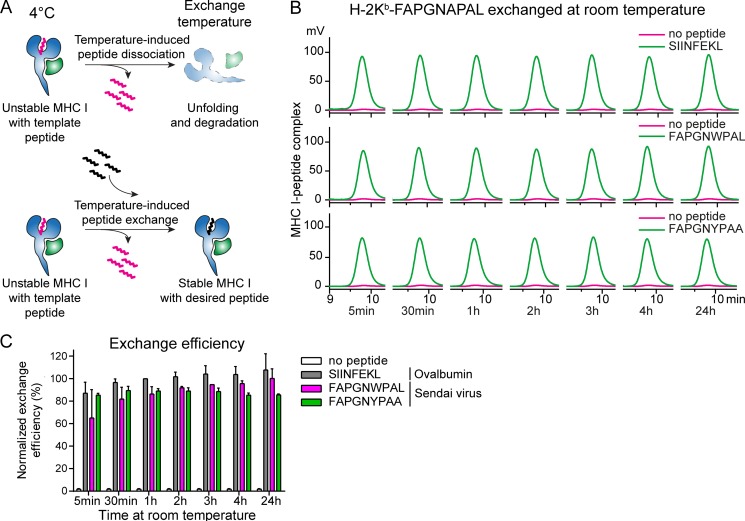
**Temperature-induced peptide exchange allows for the generation of MHC I complexes with high- and low-affinity peptides. (A)** Schematic representation of temperature-induced peptide exchange on MHC I molecules. The thermolabile MHC I–peptide complex is stable at 4°C, but undergoes unfolding and degradation under thermal challenge (upper panel). Addition of a higher-affinity peptide stabilizes the MHC I, preventing its degradation (lower panel). **(B)** Primary data of temperature-induced peptide exchange analyzed by gel filtration chromatography at room temperature. Peptide–MHC I (H-2K^b^–FAPGNAPAL) monomers were incubated with indicated peptides at room temperature over a range of time points. The following exchange peptides were used: optimal binder, SIINFEKL (ovalbumin); suboptimal binders, FAPGNWPAL or FAPGNYPAA. One of three representative experiments is shown. **(C)** The exchange efficiency was calculated from the area under the curve measured by HPLC and normalized to binding of the optimal peptide SIINFEKL for 1 h. Mean values ± SD from three independent experiments are shown.

When designing peptides suitable for MHC I temperature exchange, the most important criterion is that the MHC I complex loaded with a conditional ligand should be stable at low temperatures, but unstable at higher temperatures for replacement by exogenous peptides (Fig. 1 A). The main determinant for MHC I-peptide stability is peptide off-rate (Garstka et al., 2015). We have selected peptides known to bind to the respective MHC I molecules with low off-rates and substituted their anchor residues to increase their off-rates.

We have previously produced mouse H-2K^b^ complexes with low-affinity peptides derived from Sendai virus epitope FAPGNYPAL (NP_324–332_) and analyzed their stability and kinetics of peptide binding (Garstka et al., 2015). From the seven peptides tested, only FAPGN**A**PAL (boldface indicates amino acid changes compared to wild-type sequence) fulfilled the criteria required for peptide exchange. The melting temperature of the H-2K^b^ complex with FAPGN**A**PAL, defined as the midpoint of thermal denaturation, is ∼33°C (Fig. S1). In line with this, FAPGN**A**PAL swiftly dissociated from and did not rebind to H-2K^b^ at either of the two elevated temperatures tested (26°C and 32°C; Garstka et al., 2015). This indicates that the H-2K^b^–FAPGN**A**PAL complex is sufficiently stable to refold at 4°C, but unstable at elevated temperatures and could therefore be a suitable complex for temperature-induced peptide exchange.

To translate the exchange technology to human applications, we set out to identify a suitable peptide for HLA–A*02:01, the most frequently occurring human MHC I allele in the Caucasian population. We designed peptides based on the HIV-1 epitope ILKEPVHGV (RT_476–484_) with one (I**A**KEPVHGV or ILKEPVHG**A**) or both anchors (I**A**KEPVHG**A**) modified. HLA–A*02:01 complexes with modified peptides were produced and thermal stability experiments performed, where tryptophan fluorescence was monitored over a wide temperature range to assess HLA–A*02:01–peptide complex unfolding. Out of four complexes tested, HLA–A*02:01–I**A**KEPVHGV showed the lowest melting temperature (∼38°C; Fig. S1). This melting temperature is lower than that of the HLA–A*02:01–antigen complexes (which is around 57°C, Fig. S1), providing a temperature window for exchange from the HLA–A*02:01–I**A**KEPVHGV template.

### Temperature-labile peptide–MHC I monomers efficiently exchange for a range of peptides

We next evaluated the peptide exchange efficiency of H-2K^b^ in complex with FAPGN**A**PAL over a temperature range using analytical size exclusion HPLC. We found that the complex itself is unstable at room temperature (20°C), resulting in denaturation and aggregation. This is illustrated by the absence of an MHC I peak when analyzed by size exclusion HPLC (Fig. 1 B, in magenta). When incubated in the presence of a high-affinity peptide (SIINFEKL, OVA_257–264_) a clear peak was observed, demonstrating that H-2K^b^ could be “rescued’’ from unfolding (Fig. 1 B, upper panel; compare green to magenta). Exchange of FAPGN**A**PAL (dissociation constant [*K_d_*] > 4 µM [Garstka et al., 2015]) for SIINFEKL (*K*_d_ = 1.4 nM [Vitiello et al., 1996]) was almost complete within 30 min. The efficiency increased only by 15% after 24 h (Fig. 1 B, upper panel; quantification in Fig. 1 C, gray bar).

Similarly, HLA-A*02:01 in complex with any of the four peptides based on ILKEPVHGV were tested for exchange with a high-affinity binding peptide (vaccinia virus [VACV] B19R_297–305_; *K*_d_ = 0.06 nM [Ishizuka et al., 2009]) at different temperatures and time points. HLA-A*02:01 in complex with ILKEPVHGV or ILKEPVHG**A** remained stable at room temperature. Even at elevated temperatures, intact HLA-A*02:01 could be detected (in magenta, 37 or 42°C; Fig. S2, A and B). Considering their high melting temperatures (∼57 and 47°C, respectively; Fig. S1) and dissociation constants (ILKEPVHGV − *K*_d_ = 2.5 nM [Madden et al., 1993]; ILKEPVHG**A** − *K*_d_ = 1.1 µM, predicted with NetMHC [Nielsen et al., 2003; Andreatta and Nielsen, 2016]), ILKEPVHGV and ILKEPVHG**A** fail as input peptides in the exchange reaction.

We continued the search for optimal peptides binding to HLA–A*02:01, allowing efficient temperature-induced exchange. Complexes of HLA–A*02:01 with I**A**KEPVHGV (*K*_d_ = 7.3 µM predicted with NetMHC [Nielsen et al., 2003; Andreatta and Nielsen, 2016]) or I**A**KEPVHG**A** (*K*_d_ = 19.1 µM predicted with NetMHC [Nielsen et al., 2003; Andreatta and Nielsen, 2016]) were considerably less stable, even at room temperature (Fig. S2, C and D). As a result of higher stability, the refolding efficiency of HLA–A*02:01–I**A**KEPVHGV (at 4°C) was substantially higher than that of HLA–A*02:01–I**A**KEPVHG**A** (Fig. S2), as was maximum rescue with exogenous peptide WLIGFDFDV (Fig. S2, C and D; compare green to magenta). HLA–A*02:01–I**A**KEPVHGV was efficiently exchanged at two temperatures: at 37°C for 1 h or at 32°C for 3 h (Fig. S2 C; compare green to magenta). We selected HLA–A*02:01–I**A**KEPVHGV as the best candidate complex for peptide exchange applications, despite its higher temperature required for optimal exchange. In conclusion, we have identified two peptide–MHC I pairs allowing efficient temperature-induced exchange reactions.

When used for broad applications in immunology, MHC I multimers should exchange their peptides for numerous different peptides, including those with a relatively low affinity, including many tumor neoantigens (Duan et al., 2014). To test this, we exchanged H-2K^b^–FAPGN**A**PAL for either FAPGN**W**PAL (*K*_d_ = 33 nM at 26°C and *K*_d_ = 33 nM at 32°C [Garstka et al., 2015]) or FAPGNYPA**A** (*K*_d_ = 18 nM at 26°C and *K*_d_ = 144 nM at 32°C [Garstka et al., 2015]). For both suboptimal peptides, the exchange efficiency reached 80–90% of the level observed for SIINFEKL (Fig. 1 B; quantified in Fig. 1 C), as further confirmed by mass spectrometry (MS) analysis (Table 1). After exchange the conditional peptide FAPGN**A**PAL could not be detected, which demonstrates that all peptide–MHC I complexes contained the exogenous peptide.

**Table 1.** Relative quantification of exchange efficiency by MS

MHC I allele	MHC I monomer folded with	Template peptide exchanged for	Efficiency of exchange
			*%*
H-2K^b^	FAPGNAPAL	SIINFEKL	105.5 ± 4.7
		FAPGNWPAL	94.2 ± 10.8
		FAPGNYPAA	84.4 ± 6.2
		FAPGNAPAL	4.2 ± 0.1
		-	0.1 ± 0.1
	SIINFEKL	-	107.4 ± 12.6
HLA-A*02:01	IAKEPVHGV	NLVPMVATV	101.3 ± 13.2
		LLDQLIEEV	86.0 ± 14.6
		GLCTLVAML	70.7 ± 16.3
		IAKEPVHGV	27.4 ± 2.7
		-	7.2 ± 2.2
	NLVPMVATV	-	80.5 ± 15.3

Peptide exchange on MHC I was performed with 0.5 µM monomers (H-2K^b^ or HLA-A*02:01), incubated with 50 µM peptide as described in Materials and methods. Monomers were also incubated in the absence of peptide to determine the stability of the complexes under these conditions. To quantify the amount of eluted peptide, standard curves were created with the respective synthetic peptides. H-2K^b^–SIINFEKL and HLA-A*02:01–NLVPMVATV were measured as positive controls.

### Detection of antigen-specific CD8^+^ T cells using ready-to-use temperature-exchanged MHC I multimers

**Figure 2.**
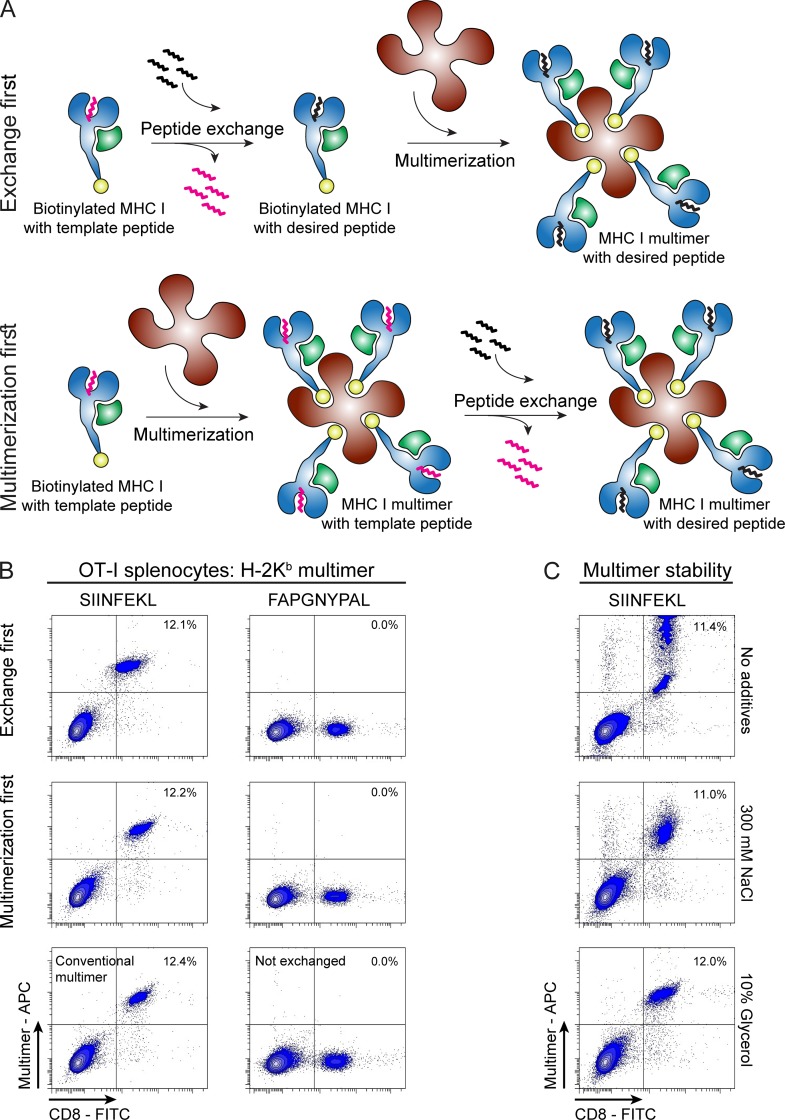
**Temperature-exchanged H-2K^b^ multimers efficiently stain antigen-specific CD8^+^ T cells. (A)** Schematic representation of MHC I peptide exchange on monomers (exchange first) or on multimers (multimerization first). **(B)** Dot plots of MHC I multimer staining of splenocytes from OT-I mice analyzed by flow cytometry. Multimers were prepared after or before exchanging the template peptide for either a relevant peptide (SIINFEKL, OVA) or an irrelevant peptide (FAPGNYPAL, Sendai virus) for 30 min at room temperature. Control multimers were prepared using conventional refolding followed by multimerization. One of three representative experiments is shown. **(C)** Thermolabile multimers of H-2K^b^–FAPGNAPAL are stable over time when stored at -80°C in the presence of 300 mM NaCl or 10% glycerol. H-2K^b^–FAPGNAPAL multimers were thawed and FAPGNAPAL was exchanged for SIINFEKL before staining OT-I splenocytes (performed once). Multimer^+^ CD8^+^ T cells are depicted as percentage of total live single cells. The gating strategy is described in detail in Fig. S4 A.

The technology of peptide exchange would be more attractive if it could be applied directly on ready-made MHC I multimers, a severe limitation of current parallel exchange technologies. In current exchange technologies, monomers are first exchanged and then multimerized (Fig. 2 A, upper panel), but the method described here can be applied directly to multimers (Fig. 2 A, lower panel). To test this, we incubated H-2K^b^-FAPGNAPAL multimers, stored batch-wise at −80°C, at room temperature either with or without 50 µM SIINFEKL peptide. After 5 min following incubation, the multimers were used to stain SIINFEKL-specific OT-I T cells. Multimers prepared by temperature exchange performed indistinguishably from conventional multimers. No positive staining was observed when multimers were not exchanged or exchanged for an irrelevant peptide (FAPGNYPAL, Fig. 2 B). When assessing multimer stability upon freezing, we found that multimers alone suffered from freeze–thaw cycles, but addition of 300 mM NaCl or 10% glycerol before freezing, as published before (Hadrup et al., 2015), ensured stability during freeze–thaw cycles (Fig. 2 C). We conclude that temperature-mediated peptide exchange can be used to produce MHC multimers with antigenic peptides from temperature-exchangeable multimer stocks within minutes. This represents a significant advantage by taking away any time-consuming preparation preceding multimer staining experiments.

**Figure 3.**
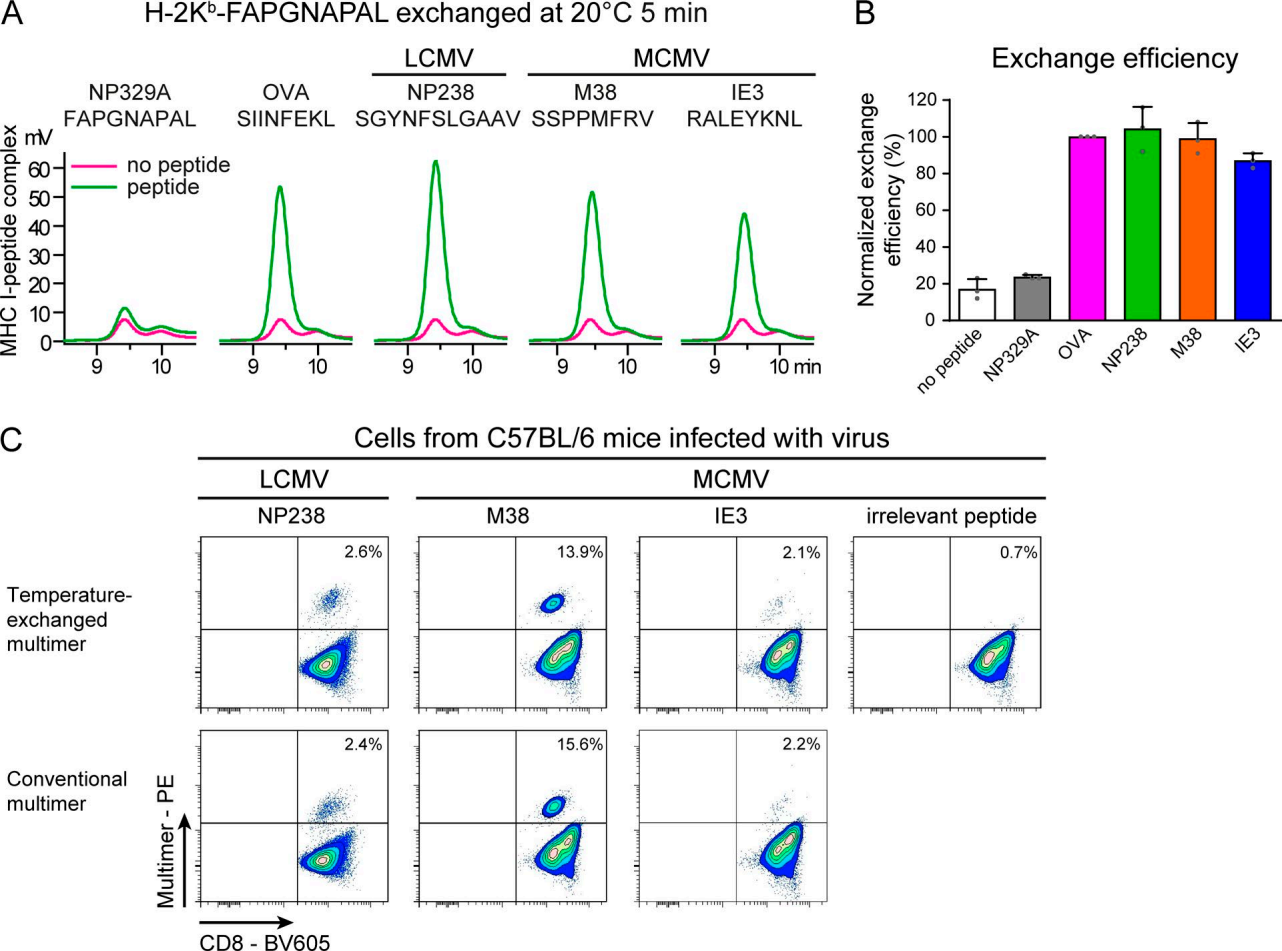
**Temperature-exchanged H-2K^b^ multimers are suitable for staining antigen-specific T cells from virus-infected mice. (A–C)** H-2K^b^–FAPGNAPAL monomers (A and B) or multimers (C) were exchanged for FAPGNAPAL (Sendai virus), SIINFEKL (OVA), SGYNFSLGAAV (LCMV NP238), SSPPMFRV (MCMV M38), or RALEYKNL (MCMV IE3) for 5 min at 20°C. **(A)** Primary data of temperature-induced peptide exchange on H-2K^b^ monomers analyzed by analytical gel filtration chromatography at room temperature. One of three representative experiments is shown. **(B)** Exchange efficiency calculated from the area under the curve from HPLC chromatograms normalized to the binding of optimal peptide (SIINFEKL). Mean values ± SD from three independent experiments (single data points depicted as gray dots) are shown. **(C)** H-2K^b^–FAPGNAPAL multimers were exchanged for the indicated peptides and subsequently used to stain corresponding CD8^+^ T cells in PBMCs of an LCMV-infected mouse or splenocytes from an MCMV-infected mouse. Percentages of CD8^+^ T cells detected by flow cytometry were comparable between temperature-exchanged multimers and conventional multimers. Irrelevant peptide: FAPGNYPAL (Sendai virus). One of two representative experiments is shown. Multimer^+^ CD8^+^ T cells are indicated as percentage of total CD8^+^ cells. Cells were gated as described in Fig. S4 B.

The immune responses to lymphocytic choriomeningitis virus (LCMV) and mouse CMV (MCMV) infections in C57BL/6 mice have been extensively characterized, and we used these infections as a model to illustrate the quality of our temperature-exchanged multimers in the detection of antigen-specific CD8^+^ T cells (Matloubian et al., 1994; van der Most et al., 1998; Rodriguez et al., 2001; Wherry et al., 2003). We measured the CD8^+^ T cell responses to the following immunodominant epitopes: LCMV epitope NP238-K^b^/SGYNFSLGAAV and MCMV epitopes M38-K^b^/SSPPMFRV and IE3-K^b^/RALEYKNL (Table S1). We first validated exchange on H-2K^b^ monomers by HPLC. As for SIINFEKL, all three peptides could be loaded with high-efficiency within 5 min at room temperature and produced stable H-2K^b^ complexes, which was not observed for exchange reactions without peptide or with an excess of high off-rate template peptide FAPGNAPAL (Fig. 3 A; quantified in Fig. 3 B). Subsequently, we again replaced the poorly H-2K^b^–binding peptide FAPGNAPAL for these three viral epitopes on H-2K^b^ multimers and used these multimers, all generated from stocks stored at −80°C as described above, to stain blood samples from LCMV-infected mice or splenocytes from MCMV-infected mice. Within 5 min after taking the multimers with temperature-sensitive peptides from the freezer, the antigenic peptide-loaded multimers were ready and stained antigen-specific CD8^+^ T cells as efficiently as conventional multimers (Fig. 3 C), demonstrating the easy and broad use of temperature exchange technology.

**Figure 4.**
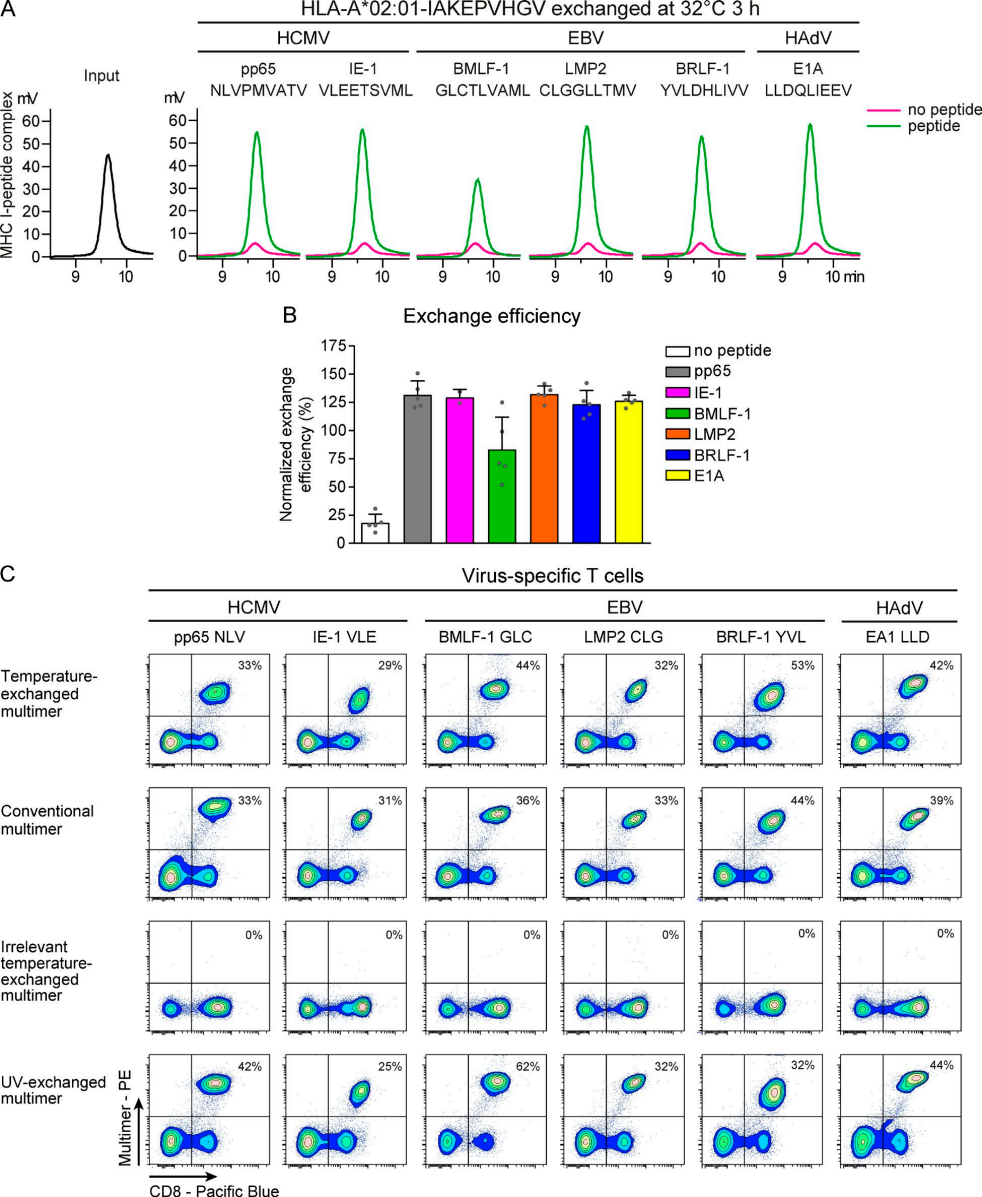
Temperature-exchanged HLA-A*02:01 multimers are suitable for staining virus-specific T cells. (A–C) HLA-A*02:01–IAKEPVHGV monomers (A and B) or multimers (C) were exchanged for HCMV pp65-A2/NLVPMVATV, HCMV IE-1-A2/VLEETSVML, EBV BMLF-1-A2/GLCTLVAML, EBV LMP2-A2/CLGGLLTMV, EBV BRLF-1-A2/YVLDHLIVV, or HAdV E1A-A2/LLDQLIEEV for 3 h at 32°C. **(A)** Representative chromatograms of exchange on monomers analyzed by gel filtration chromatography at room temperature. **(B)** Efficiency of exchange calculated from the area under the curve from HPLC chromatograms normalized to input peptide–MHC I. Mean values ± SD from five independent experiments are shown. Single data points are depicted as gray dots. **(C)** HLA-A*02:01–IAKEPVHGV multimers were exchanged for the indicated peptides and subsequently used for staining of specific CD8^+^ T cell clones or cell lines. Detected percentages of multimer-positive CD8^+^ T cells were comparable between temperature-exchanged multimers and conventional multimers. One of two representative flow cytometry experiments is shown. Multimer^+^ CD8^+^ T cells are indicated as percentage of total CD8^+^ cells. Cells were gated as described in Fig. S4 C.

Likewise, HLA–A*02:01–IAKEPVHGV monomers could be readily exchanged for selected viral epitopes (HCMV pp65-A2/NLVPMVATV, HCMV IE-1-A2/VLEETSVML, EBV LMP2-A2/CLGGLLTMV, EBV BMLF-1-A2/GLCTLVAML, EBV BRLF1-A2/YVLDHLIVV, and human adenovirus [HAdV] E1A-A2/LLDQLIEEV [details in Table S1]), when incubated at 32°C for 3 h or 37°C for 45 min (Fig. 4 and Fig. S3). HPLC analysis revealed no MHC I peak after incubation at 32°C without peptide, indicating unfolding and precipitation of MHC I monomers (Fig. 4 A, magenta). However, incubation with peptide at 32°C for 3 h revealed a peak of MHC I monomers as high as the original input complexes for all peptides (Fig. 4 A; quantified in Fig. 4 B). Incubation at 37°C for 45 min likewise resulted in efficient rescue, with the exception of EBV BMLF-1-A2/GLC (Fig. S3). Considering the relatively low predicted affinity of this epitope (*K*_d_ = 138.63 nM predicted with NetMHC [Nielsen et al., 2003; Andreatta and Nielsen, 2016]), EBV BMLF-1-A2/GLC may not stabilize HLA-A2*02:01 sufficiently at elevated temperatures during a prolonged period of time. We selected 3-h incubation with exogenous peptides at 32°C as optimal exchange condition for HLA–A*02:01. MS analysis showed that HLA–A*02:01–IAKEPVHGV monomers exchanged for NLVPMVATV, LLDQLIEEV, GLCTLVAML, or template peptide IAKEPVHGV contained only the desired peptides. The rescue of the MHC I monomers was proportional to the predicted affinity of the peptides, as observed in the HPLC quantifications (Table 1 and Fig. 4 B).

Within 3 h after addition of peptide to the preformed conditional HLA–A*02:01, multimers they were ready for staining of CD8^+^ T cell clones with corresponding specificities. Detected percentages of multimer-positive CD8^+^ T cells corresponded to those detected using either conventional or UV-exchanged multimers, confirming their proper function. No staining was observed with multimers exchanged for irrelevant peptides (Fig. 4 C).

### Exchanged MHC I-peptide multimers are effective reagents for immune monitoring

To demonstrate a direct application of our reagents in clinical practice, we compared our temperature-exchanged multimers with conventional multimers in an immune monitoring setting. Because patients are heavily immunocompromised after T cell–depleted allogeneic stem cell transplantation (allo-SCT), T cell reconstitution is critical to prevent morbidity and mortality caused by posttransplant infections with herpesviruses like human CMV (HCMV) and EBV (Broers et al., 2000; Green et al., 2016). Therefore, patients are intensively monitored until the donor-derived immune system has developed. Ready-to-use multimers are valuable immune monitoring reagents that allow prompt action as needed in these cases.

**Figure 5.**
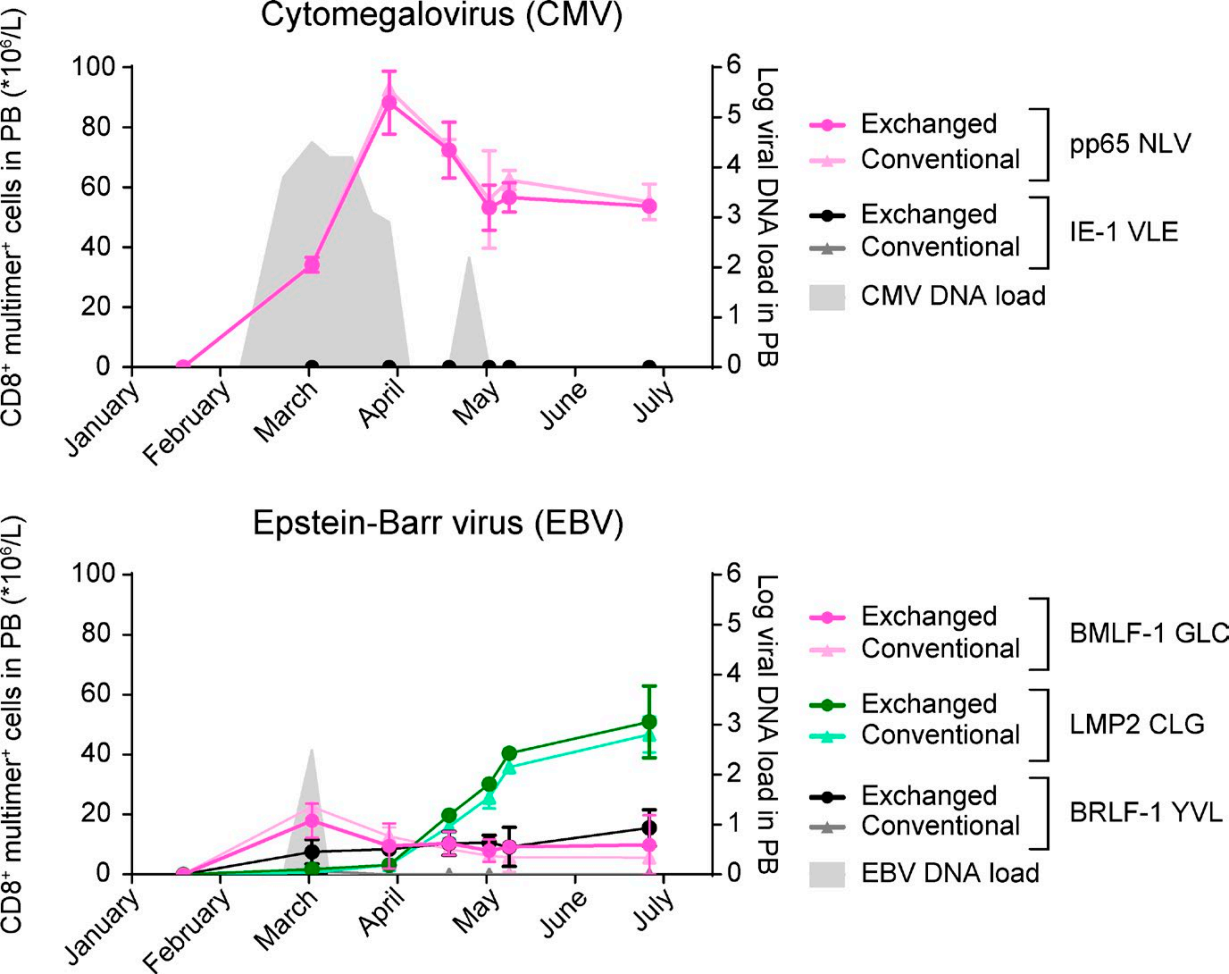
Temperature-exchanged HLA-A*02:01 multimers can be used for monitoring of HCMV- and EBV-specific CD8^+^ T cells in peripheral blood of an allo-SCT recipient. Peripheral blood (PB) samples taken after allo-SCT were analyzed for virus-specific CD8^+^ T cells in relation to viral DNA loads (gray). The frequency of HCMV- and EBV-specific T cells within the CD8^+^ T cell populations was determined using temperature-exchanged (dark colors) and conventional (light colors) MHC I multimer staining analyzed by flow cytometry. Mean values ± SD from two experiments performed on the same day are shown.

We exchanged PE-labeled HLA–A*02:01–IAKEPVHGV multimers (stored at −80°C and exchanged following the conditions as described above) for a selection of HCMV- and EBV-derived epitopes in parallel and used these to monitor T cell frequencies in peripheral blood mononuclear cells (PBMCs) obtained at weekly intervals after allo-SCT. The kinetics of CD8^+^ T cells specific for HCMV pp65-A2/NLV are in concordance with the HCMV reactivation illustrated by the expansion of HCMV viral DNA detected in blood (Fig. 5, upper panel). Although a positive EBV DNA load was measured only once, CD8^+^ T cells specific for EBV LMP2-A2/CLG, and to a lesser extent those specific for EBV BMLF-1-A2/GLC, expanded over time (Fig. 5, lower panel). No significant responses were detected against HCMV IE-1-A2/VLE (Fig. 5, upper panel) or EBV BRLF1-A2/YVL (Fig. 5, lower panel). Frequencies of specific T cells were comparable between conventional and temperature-exchanged multimers. This illustrates the efficiency and flexibility of our technology to rapidly produce many different MHC I multimers ad hoc from a stored and ready-to-use stock for the detection of antigen-specific T cells, even at the low frequencies typically found in primary immune monitoring samples.

## Discussion

We describe a reliable approach that allows the parallel generation of large sets of different MHC I multimers. Our approach can be applied in all laboratories, because it requires only a freezer for storage of exchangeable multimer stocks and a thermoblock, water bath, or PCR machine for incubation at the optimal temperature for exchange. This system is faster and less laborious than the generation of multimers from single peptide–MHC I combinations, either made by producing individual complexes by refolding and purification or by cleaving an MHC-embedded peptide for chemically triggered or UV-mediated peptide exchange (Rodenko et al., 2006, 2009; Amore et al., 2013; Choo et al., 2014). Our approach allows fast and near-quantitative peptide exchange on multimers, whereas parallel multimer generation using UV-mediated exchange is variable as a result of uneven evaporation across and between sample plates and cannot be performed on preformed MHC I multimers because of fluorophore bleaching. We have established a method where ready-made temperature-sensitive MHC I multimers can be stored at −80°C and, while thawing, can be ad hoc incubated with peptides of choice to allow peptide exchange within 5–180 min, depending on the MHC I allele. This is the most robust technique for multimer production developed to date, that will facilitate immune monitoring and discovery of neoantigens. We anticipate that rapid, robust, and inexpensive detection of MHC antigen–specific T cells will have a strong impact on the immune monitoring of responses to infection and cancer immunotherapies, as well as vaccines (Bentzen et al., 2016; El Bissati et al., 2016; Grassmann et al., 2017; La Rosa et al., 2017). Immunotherapy, aimed at either suppressing or enhancing cellular immune responses, has advanced greatly over the last decade. Several immune checkpoint inhibitors, including antibodies against CTLA-4 and PD-1/PD-L1, have been approved for use in the clinic and have shown remarkable responses in the treatment of various cancers, including melanoma, non–small cell lung cancer and renal cell cancer (Hodi et al., 2010; Page et al., 2010; Topalian et al., 2012; Robert et al., 2014; Tumeh et al., 2014). As a consequence of checkpoint blockade, T cell responses elicited against neoantigens are markedly increased, leading to improved killing of cancer cells (Fourcade et al., 2009; van Rooij et al., 2013). A combination of therapies directed at immune checkpoints and the information in the cancer mutanome holds great promise in personalized cancer treatment. Identifying T cell responses against neoantigens and other cancer-specific epitopes will contribute to the success of immunotherapy, especially when combined with vaccination.

We have shown for two MHC I alleles, one mouse and one human, that temperature-exchanged multimers can be as efficient as conventional- or UV-exchanged multimers to stain specific CD8^+^ T cells, including those present at low frequencies. We have demonstrated for both the H-2K^b^–FAPGNAPAL and HLA–A*02:01–IAKEPVHGV combinations that the temperature-labile input peptide may be exchanged for both high- and low-affinity peptides, illustrating the application for a broad array of T cell specificities (Fig. 1, B and C; Fig. 3; Fig. 4; and Table S1). These MHC I multimers loaded with desired peptides are highly specific, as no difference in background stain as compared with conventional or UV-exchanged multimers was observed (Fig. 2; Fig. 3; and Fig. 4). Their use in monitoring viral reactivation in an allo-SCT recipient illustrates the flexibility of temperature-exchangeable MHC I multimers that can be produced within hours, as required for clinical use (Fig. 5).

We previously showed that peptide–MHC I complexes at a given temperature undergo a conformational change, which results in full peptide dissociation (Garstka et al., 2015). Below this temperature, the complexes are fairly stable and, as a result of a high off-rate, allow exchange for a more stable low off-rate peptide. We designed peptides to form stable complexes with MHC I at low temperatures that can be released at elevated temperatures. The selection of optimal peptides allowing low temperature exchange and full replacement by exogenous peptides is not obvious. Several options include peptides with suboptimal length, smaller anchor residues, and altered N or C termini (Garstka et al., 2015). Even then, many peptide sequences have to be tested to identify the optimal peptide–MHC I combination, as we describe here for the most frequently used mouse and human MHC I alleles. The design of peptides suitable for temperature exchange on HLA–A*02:01 proved more challenging than H-2K^b^, possibly because of the intrinsically higher stability of human MHC I complexes compared with mouse MHC I. Yet, expanding this principle to the many other MHC I alleles could provide a standard procedure where viral or tumor antigens are sequenced, the fragments that may bind are predicted and synthesized within a day, and loaded on the ready-to-use MHC I multimers (as stored in the −80°C freezer). Within 2 d a patient’s T cell responses could then be monitored, as the production of the MHC I multimers loaded with the correct peptides is no longer the time limiting factor.

In conclusion, we present a fast, easy, and reproducible method for the generation of ready-to-use MHC I multimers loaded with epitopes at wish. This approach will render MHC multimer technology accessible to any research or clinical chemistry laboratory.

## Materials and methods

### Peptide synthesis and purification

Peptides were synthesized in our laboratory by standard solid-phase peptide synthesis in *N*-methyl-2-pyrrolidone using Syro I and Syro II synthesizers. Amino acids and resins were used as purchased from Nova Biochem. Peptides were purified by reversed-phase HPLC using a Waters HPLC system equipped with a preparative Waters X-bridge C18 column. The mobile phase consisted of water/acetonitrile mixtures containing 0.1% TFA. Peptide purity and composition were confirmed by LC-MS using a mass spectrometer (Micromass LCT Premier; Waters) equipped with a 2795 separation module (Alliance HT) and photodiode array detector (2996; Waters Chromatography). LC-MS samples were run over a Kinetex C18 column (Phenomenex) in a water/acetonitrile gradient. Analysis was performed using MassLynx 4.1 software (Waters Chromatography). Peptides were purified twice if necessary.

### Protein expression and purification

MHC I complexes were expressed and refolded according to previously published protocols (Toebes et al., 2009). Refolded complexes of H-2K^b^ were purified twice using anion exchange (0–1 M NaCl in 20 mM Tris-HCl, pH 8; Resource Q column) and size exclusion chromatography (150 mM NaCl and 20 mM Tris-HCl, pH 8; Superdex 75 16/600 column) on an ÄKTA (GE Healthcare Life Sciences) or NGC system (Bio-Rad). We discovered that recovery was considerably lower when purifying using anion exchange and size exclusion chromatography, as compared with using size exclusion only, possibly caused by strong interaction between peptide and ion-exchange resin. Therefore, to maximize purification yields, refolded complexes of HLA–A*02:01 were purified using only size exclusion chromatography (300 mM NaCl and 20 mM Tris-HCl, pH 8). Purified properly folded complexes were concentrated using Amicon Ultra-15 30 kD MWCO centrifugal filter units (Merck Millipore), directly biotinylated using BirA ligase where needed, purified again using size exclusion chromatography and stored in 300 mM NaCl and 20 mM Tris-HCl, pH 8.0 with 15% glycerol at −80°C until further use.

### Protein unfolding

Thermal unfolding of different H-2K^b^– and HLA–A*02:01–peptide complexes was determined using an Optim 1000 (Avacta Analytical) machine. Peptide–MHC I complexes were measured in 150 mM NaCl and 20 mM Tris-HCl, pH 7.5, or PBS at a protein concentration of 0.2 mg/ml. Samples were heated using a 1°C step gradient with 30 s temperature stabilization for each step. Unfolding was followed by measuring tryptophan fluorescence emission at a range from 300 to 400 nm after excitation at 266 nm. Barycentric fluorescence was determined according to the equation

BCMλ=(∑I[λ]×λ)/(∑I[λ])

where BCMλ is the Barycentric mean fluorescence in nm, I(λ) is the fluorescence intensity at a given wavelength, and λ is the wavelength in nm.

The melting temperature (T_m_) was calculated using Barycentric fluorescence as a function of temperature according to the equation

$$T_{m}=max\frac{dBMC}{dt}\left(T\right)$$

where max is the local maximum and $\frac{dBCM}{dt}\left(T\right)$ is the first derivative of Barycentric fluorescence as a function of temperature in $\left[\frac{nm}{{}^{\circ}C}\right]$.

Analysis was performed with Optim Analysis Software v 2.0 (Avacta Analytical).

### Multimerization of MHC I monomers

MHC I monomers were complexed with allophycocyanin (APC)- or PE-labeled streptavidin to form multimers for T cell analysis. Typically, temperature-labile peptide–MHC I complexes were multimerized on ice by stepwise addition of fluorochrome-labeled streptavidin with 1-min intervals. Full biotinylation was verified by HPLC. Aliquots of multimers were snap frozen in 150 mM NaCl and 20 mM Tris-HCl, pH 7.5, containing 15% glycerol.

### HPLC analysis of temperature-mediated peptide exchange

To initiate peptide exchange, 0.5 µM peptide–MHC I complex was incubated with 50 µM exchange peptide in 110 µl PBS under defined exchange conditions. After incubation exchange solutions were centrifuged at 14,000 *g* for 1 min at room temperature, and subsequently, the supernatant was analyzed by gel filtration on a Shimadzu Prominence HPLC system equipped with a 300 × 7.8 mm BioSep SEC–s3000 column (Phenomenex) using PBS as mobile phase. Data were processed and analyzed using Shimadzu LabSolutions software (version 5.85).

### Relative exchange efficiency determined by MS

To quantify peptide exchange on H-2K^b^, 0.5 µM H-2K^b^ monomers (H-2K^b^–FAPGNAPAL) were incubated with 50 µM peptide (SIINFEKL, FAPGNWPAL, FAPGNYPAA, or FAPGNAPAL) in PBS for 45 min at room temperature. For quantification of peptide exchange on HLA–A*02:01, 0.5 µM HLA–A*02:01 monomers were incubated with 50 µM peptide in PBS for 3 h at 32°C.

Before analysis, exchanged monomers were spun at 14,000 *g* for 1 min at room temperature to remove aggregates and subsequently purified using a Microcon 30-kD Centrifugal Filter Unit with Ultracel-30 membrane (Merck Millipore; preincubated with tryptic BSA digest to prevent stickiness of the peptides to the membrane) to remove unbound excess peptide. After washing twice with PBS and twice with ammonium bicarbonate at room temperature, MHC-bound peptides were eluted by the addition of 200 µl of 10% acetic acid followed by mixing at 600 rpm for 1 min at room temperature. Eluted peptides were separated using a Microcon 30-kD Centrifugal Filter Unit with Ultracel-30 membranes. Eluates were lyophilized and subjected to MS analysis.

For MS analysis, peptides were dissolved in 95/3/0.1 vol/vol/vol water/acetonitrile/formic acid and subsequently analyzed by on-line nano-HPLC MS/MS using a 1100 HPLC system (Agilent Technologies), as described previously (Meiring et al., 2002). Peptides were trapped at 10 µl/min on a 15-mm column (100-μm inner diameter [ID]; ReproSil-Pur C18-AQ, 3 µm, Dr. Maisch GmbH) and eluted to a 200-mm column (50-μm ID; ReproSil-Pur C18-AQ, 3 µm) at 150 nl/min. All columns were packed in house. The column was developed with a 30-min gradient from 0 to 50% acetonitrile in 0.1% formic acid. The end of the nano-LC column was drawn to a tip (5-µm ID), from which the eluent was sprayed into a 7-tesla LTQ-FT Ultra mass spectrometer (Thermo Electron). The mass spectrometer was operated in data-dependent mode, automatically switching between MS and MS/MS acquisition. Full scan MS spectra were acquired in the Fourier-transform ion cyclotron resonance (FT-ICR) with a resolution of 25,000 at a target value of 3,000,000. The two most intense ions were then isolated for accurate mass measurements by a selected ion-monitoring scan in FT-ICR with a resolution of 50,000 at a target accumulation value of 50,000. Selected ions were fragmented in the linear ion trap using collision-induced dissociation at a target value of 10,000. To quantify the amount of eluted peptide standard curves were created with the respective synthetic peptides.

### Mice

Wild-type C57BL/6 mice (Charles River) were maintained at the Central Animal Facility of the Leiden University Medical Center (LUMC) under specific pathogen-free conditions. Mice were infected intraperitoneally with 5 × 10^4^ PFU MCMV-Smith (VR-194; American Type Culture Collection), derived from salivary gland stocks from MCMV-infected BALB/c mice, or with 2 × 10^5^ PFU LCMV Armstrong propagated on baby hamster kidney cells. Virus titers were determined by plaque assays as published (Welten et al., 2015). All animal experiments were performed with approval of the Animal Experiments Committee of the LUMC and according to the Dutch Experiments on Animals Act that serves the implementation of Guidelines on the Protection of Experimental Animals by the Council of Europe and the guide to animal experimentation set by the LUMC.

### Collection of primary human material

Peripheral blood samples were obtained from a HLA–A*02:01–positive multiple myeloma patient after T cell–depleted allo-SCT, after approval by the LUMC and written informed consent according to the Declaration of Helsinki. To monitor viral reactivation EBV and HCMV DNA loads on fresh whole blood were assessed by quantitative PCR (qPCR). PBMCs were collected using Ficoll Isopaque separation (LUMC Pharmacy, Leiden, Netherlands) and cryopreserved in the vapor phase of liquid nitrogen. Virus-specific CD8^+^ T cell reconstitution was determined on thawed PBMCs by flow cytometry.

### Antibodies and reagents

Ficoll Isopaque was obtained from the LUMC Pharmacy (Leiden, Netherlands). Fluorochrome-conjugated antibodies were purchased from several suppliers. V500 anti-mouse CD3, FITC anti–mouse CD8, FITC anti–human CD4, Pacific Blue anti–human CD8, and APC anti–human CD14 were purchased from BD Biosciences. BV605 anti–mouse CD8 was purchased from BioLegend. Fluorochrome-conjugated streptavidin and 7-AAD were purchased from Invitrogen. DAPI was purchased from Sigma. Conventional HLA–A*02:01 PE-labeled tetramers were produced by K.L.M.C. Franken, M.G.D. Kester, and L. Hageman (LUMC, Leiden, Netherlands) as described previously (Altman et al., 1996). Human IL-2 was purchased from Chiron. Human serum albumin was purchased from Sanquin Reagents.

### Flow cytometry analysis of mouse CD8^+^ T cells

H-2K^b^–FAPGNAPAL multimers were exchanged for selected peptides for 5 min at room temperature and subsequently used for staining of the H-2K^b^–restricted OVA_257–264_–specific TCR transgenic line (OT-I), described previously (Hogquist et al., 1994). Generally, 200,000 cells were stained first with APC- or PE-labeled temperature-exchanged or conventional multimers for 10 min at room temperature and then with surface marker antibodies (anti–CD8-FITC) at 4°C for 20 min. Cells were washed twice with and then resuspended in FACS buffer (0.5% BSA and 0.02% sodium azide in PBS). DAPI was added at a final concentration of 0.1 µg/ml. Samples were measured using a flow cytometer (FACSAria Fusion; BD Biosciences) and data were analyzed with FACSDiva software (version 8.0.2; BD Biosciences; gating strategy in Fig. S4).

Virus-specific T cells were analyzed in blood samples of LCMV-infected mice after erythrocyte lysis or splenocytes obtained from MCMV-infected, 8–10-wk-old mice (infected at 6–8 wk). Erythrocytes were lysed using a hypotonic ammonium chloride buffer (150 mM NH_4_Cl and 10 mM KHCO_3_, pH 7.2 ± 0.2). Cells were simultaneously stained with appropriate temperature-exchanged multimers and surface markers (7-AAD, anti–CD3-V500, and anti–CD8-BV605) for 30 min at 4°C. Multimers were titrated to establish optimal T cell staining. Generally, a dilution of 1:20–1:40 was sufficient to stain 10,000–100,000 T cells in 50 µl FACS buffer. Cells were washed twice with and resuspended in FACS buffer. Sample data were acquired using a flow cytometer (Fortessa; BD Biosciences) and analyzed using FACSDiva software (version 8.0.2; BD Biosciences; gating strategy in Fig. S4).

### Flow cytometry analysis of human CD8^+^ T cells

Multimers of HLA–A*02:01–IAKEPVHGV were exchanged for selected peptides at 32°C for 3 h and used to stain corresponding CD8^+^ T cells. UV-exchanged multimers were produced and exchanged following published protocols (Rodenko et al., 2006; Toebes et al., 2006). Clones or cell lines of the indicated viral T cell specificities (cultured in IMDM supplemented with 10% human serum and 100 IU/ml IL-2) were mixed with PBMCs of a HLA–A*02:01–negative donor to be able to discriminate multimer-positive from multimer-negative cells. After incubation with PE-labeled temperature-exchanged, conventional or UV-exchanged multimers for 10 min at 4°C, cells were stained with surface marker antibodies (anti–CD8-Pacific Blue and anti–CD14-APC) for 20 min at 4°C. Multimers were titrated to establish optimal T cell staining without background. Cells were washed twice with and resuspended in FACS buffer (0.5% human serum albumin in PBS). Samples were acquired using a flow cytometer (FACSCanto II; BD) and analysis was performed with FACSDiva software (version 8.0.2; BD Biosciences; gating strategy in Fig. S4). Absolute numbers of multimer positive CD8^+^ T cells were calculated based on the percentage of multimer positive cells within the CD8^+^ T cell population and the concentration of CD8^+^ T cells in whole blood.

### Online supplemental material

Fig. S1 shows the thermal denaturation of selected peptide–MHC I complexes analyzed to determine melting temperatures. Fig. S2 demonstrates the temperature stability of HLA–A*02:01 in complex with peptides of the ILKEKVHGV series, investigated using analytical gel filtration chromatography. Fig. S3 shows that exchange of HLA–A*02:01–IAKEPVHGV at 37°C for 45 min is efficient for high-affinity peptides, but not for low-affinity peptides. Fig. S4 illustrates the gating strategies used in flow cytometry experiments. Table S1 lists all peptides and *K*_d_s mentioned in this article.

## Supplementary Material

Supplemental Materials (PDF)
