# Supplementary material for: A flexible MHC class I multimer loading system for large-scale detection of antigen-specific T cells
Source: J Exp Med. 2018 May 7;215(5):1493–504. doi: 10.1084/jem.20180156 (PMC5940271; doi:10.1084/jem.20180156)
Supplement: Supplemental Materials (PDF) [file JEM_20180156_sm.pdf]

Supplemental material

Luimstra et al., https://doi.org/10.1084/jem.20180156

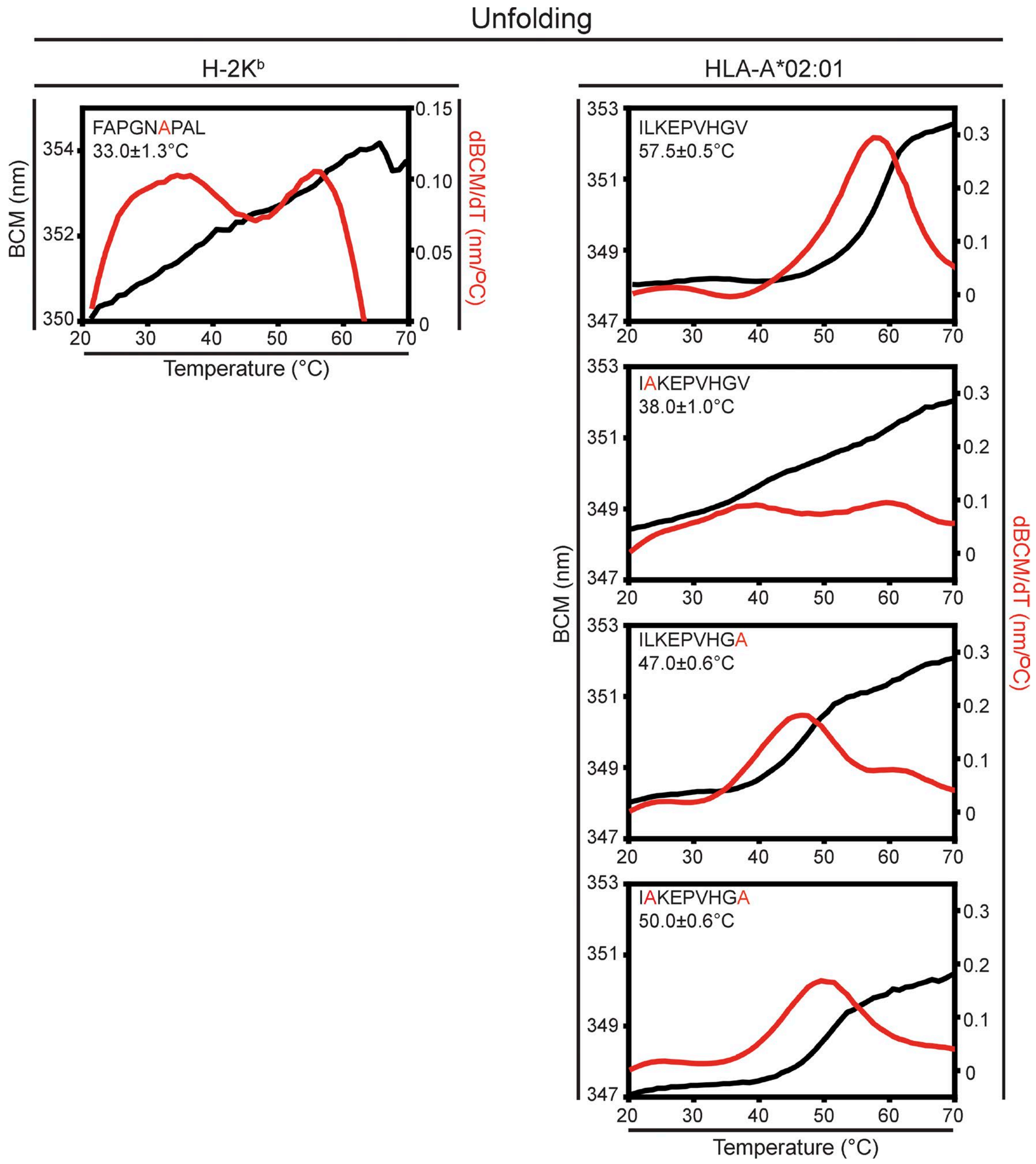

Figure S1. **Defining the temperature range for temperature-induced peptide exchange.** Thermal denaturation of peptide–MHC I complexes measured by barycentric mean fluorescence (BCM, in black). The fit to the first derivate of BCM (in red) shows the melting temperature as a maximum. Melting temperatures are mean values ± SD from four independent experiments.

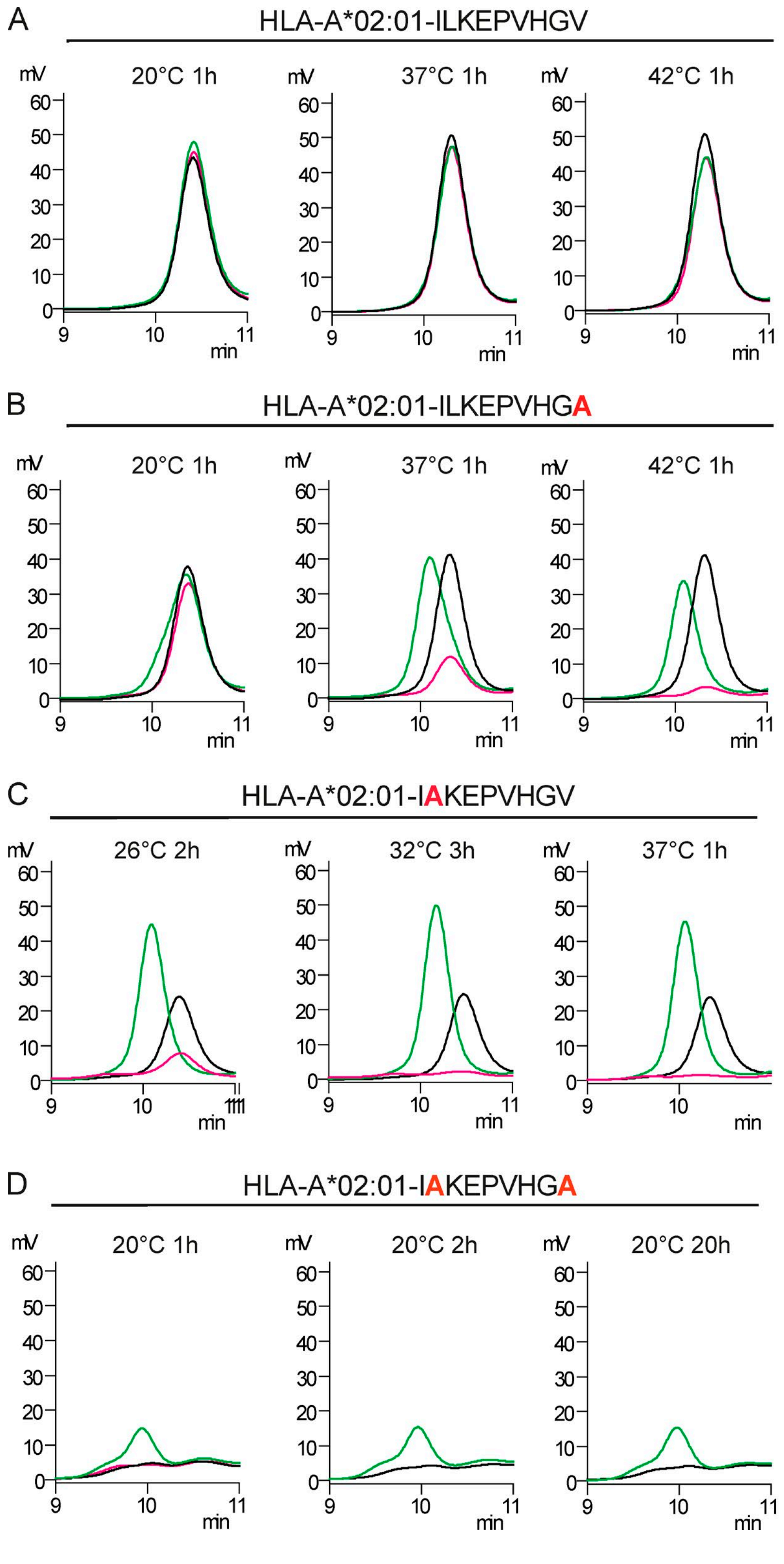

Peptide	Refolding efficiency
A - ILKEPVHGV	2.6%
B - IAKEPVHGV	5.7%
C - ILKEPVHGA	3.7%
D - IAKEPVHGA	0.9%

Figure S2. **HLA-A*02:01 in complex with IAKEPVHGV peptide is the most suitable for temperature-induced exchange.** HLA-A*02:01–ILKEPVHGV, HLA-A*02:01–ILKEPVHG**A**, HLA-A*02:01–I**A**KEPVHGV, and HLA-A*02:01–I**A**KEPVHG**A** complexes were incubated with a high affinity peptide (vaccinia virus epitope WLIGFDFDV, green line) or without peptide (magenta line) at indicated temperatures and times. HLA-A*02:01 was used at a concentration of 0.5 µM and exchange peptide was used at a concentration of 50 µM. Overlays include nonincubated template complex (input, black line) for comparison. HLA-A*02:01-ILKEPVHGV **(A)** and HLA-A*02:01-ILKEPVHG**A** **(B)** remain stable at RT, but HLA-A*02:01-I**A**KEPVHGV **(C)** and HLA-A*02:01-I**A**KEPVHG**A** **(D)** complexes are unstable at RT and are therefore suitable for exchange. Indicated refolding efficiencies are represented as a percentage of purified properly folded HLA-A*02:01–peptide complex related to input free heavy chain (from inclusion bodies).

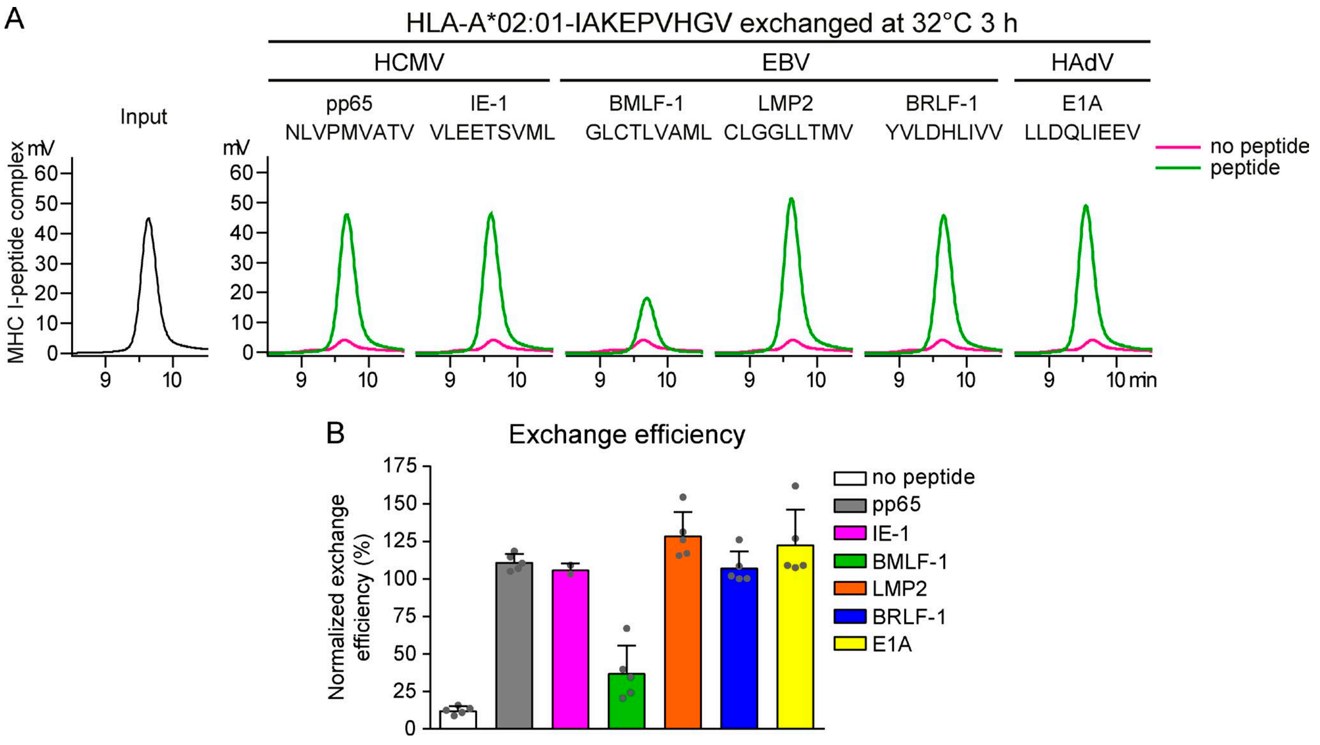

Figure S3. **Exchange of HLA-A*02:01-IAKEPVHGV at 37°C for 45 min (related to Fig. 4).** HLA-A*02:01–IAKEPVHGV monomers were incubated with HCMV pp65-A2/NLVPMVATV, HCMV IE-1-A2/VLEETSVML, EBV BMLF-1-A2/GLCTLVAML, EBV LMP2-A2/CLGGLLTMV, EBV BRLF-1-A2/YVLDHLIVV, or HAdV E1A-A2/LLDQLIEEV for 45 min at 37°C. **(A)** Exchange on monomers analyzed by gel filtration chromatography. One of five representative experiments is shown. **(B)** Efficiency of exchange calculated from the area under the curve from chromatograms normalized to the input peptide-MHC I. Mean values ± SD from five independent experiments are shown. Single data points are depicted as gray dots.

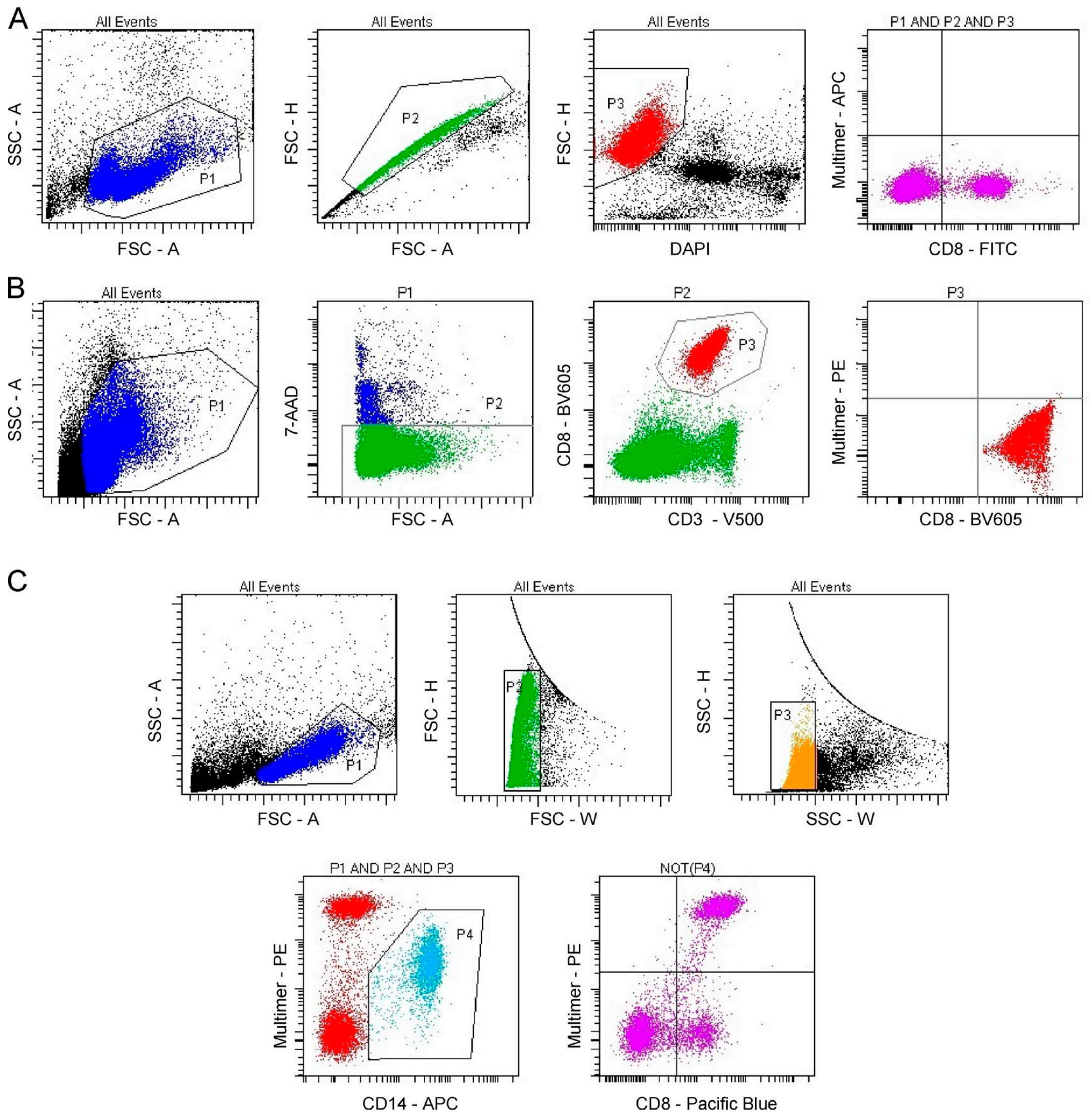

Figure S4. **Gating strategies used for flow cytometry analysis. (A)** Example of the gating strategy used to analyze OT-I cells (Fig. 2, B and C). Dead cells and debris were excluded based on forward scatter (FSC) and side scatter (SSC; P1), doublets were excluded based on FSC-H and FSC-A (P2), and live cells were gated using DAPI (P3). Cells that fell within all three of these populations were plotted with CD8 (FITC) and multimer (APC). Per sample, 20,000–55,000 events in P1 were acquired. **(B)** Example of the gating strategy used to analyze mouse cells (Fig. 3 C). Dead cells and debris were excluded based on FSC and SSC (P1), dead cells were excluded using 7-AAD (P2), and $CD8^+$ T cells were gated using anti-CD3 (V500) and anti-CD8 (BV605; P3). Cells that fell within all three of these populations were plotted with CD8 (BV605) and multimer (PE). Per sample 40,000–60,000 events were acquired. **(C)** Example of the gating strategy used to analyze human cells (Figs. 4 C and 5). Dead cells and debris were excluded based on FSC and SSC (P1), doublets were excluded based on FSC-H versus FSC-W (P2) and SSC-H versus SSC-W (P3), and monocytes were excluded using anti-CD14 (APC; P4). Cells that fall within all of these populations were plotted with CD8 (Pacific Blue) and multimer (PE). Per sample around 20,000 events in P1 were acquired.

Table S1. **Peptides used in this study and descriptions of their modifications (related to Figs. 1–5 and Figs. S1–S4 and Table 1)**

MHC I allele	Source	Epitope	Sequence	K_d
				nM
H-2K[b]	Ovalbumin	$OVA_{257-264}$	SIINFEKL	1.4 (Vitiello et al., 1996)
	Sendai virus	$NP_{324-332}$	FAPGNYPAL	108 (Garstka et al., 2015)
			FAPGN**W**PAL	31 (Garstka et al., 2015)
			FAPGNYPA**A**	144 (Garstka et al., 2015)
			FAPGN**A**PAL	>4,000 (Garstka et al., 2015)
	LCMV	$NP_{238-248}$	SGYNFSLGAAV	0.38 (Kotturi et al., 2007)
	MCMV	$M38_{316-323}$	SSPPMFRV	392* (Nielsen et al., 2003; Andreatta and Nielsen, 2016)
		$IE3_{416-423}$	RALEYKNL	23* (Nielsen et al., 2003; Andreatta and Nielsen, 2016)
HLA-A*02:01	VACV	$B19R_{294-302}$	WLIGFDFDV	0.06 (Ishizuka et al., 2009)
	HIV-1	$RT_{476-484}$	ILKEPVHGV	2.5 (Madden et al., 1993)
			I**A**KEPVHGV	7,288* (Nielsen et al., 2003; Andreatta and Nielsen, 2016)
			ILKEPVHG**A**	1,095* (Nielsen et al., 2003; Andreatta and Nielsen, 2016)
			I**A**KEPVHG**A**	19,111* (Nielsen et al., 2003; Andreatta and Nielsen, 2016)
	HCMV	$pp65_{495-503}$	NLVPMVATV	26* (Nielsen et al., 2003; Andreatta and Nielsen, 2016)
		$IE\text{-}1_{316-324}$	VLEETSVML	297* (Nielsen et al., 2003; Andreatta and Nielsen, 2016)
	EBV	$BMLF\text{-}1_{280-288}$	GLCTLVAML	139* (Nielsen et al., 2003; Andreatta and Nielsen, 2016)
		$LMP2_{426-434}$	CLGGLLTMV	76* (Nielsen et al., 2003; Andreatta and Nielsen, 2016)
		$BRLF\text{-}1_{109-117}$	YVLDHLIVV	4.1* (Nielsen et al., 2003; Andreatta and Nielsen, 2016)
	HAdV	$E1A_{19-27}$	LLDQLIEEV	16* (Nielsen et al., 2003; Andreatta and Nielsen, 2016)

Some of the peptides used are derivatives of FAPGNYPAL or ILKEPVHGV modified at anchor positions (indicated in bold). M, mouse; H, human. Affinity to the respective MHC is either from published evidence or predicted with NetMHC (indicated with *).

References

Andreatta, M., and M. Nielsen. 2016. Gapped sequence alignment using artificial neural networks: application to the MHC class I system. *Bioinformatics.* 32:511–517. https://doi.org/10.1093/bioinformatics/btv639

Garstka, M.A., A. Fish, P.H. Celie, R.P. Joosten, G.M. Janssen, I. Berlin, R. Hoppes, M. Stadnik, L. Janssen, H. Ovaa, et al. 2015. The first step of peptide selection in antigen presentation by MHC class I molecules. *Proc. Natl. Acad. Sci. USA.* 112:1505–1510. https://doi.org/10.1073/pnas.1416543112

Ishizuka, J., K. Grebe, E. Shenderov, B. Peters, Q. Chen, Y. Peng, L. Wang, T. Dong, V. Pasquetto, C. Oseroff, et al. 2009. Quantitating T cell cross-reactivity for unrelated peptide antigens. *J. Immunol.* 183:4337–4345. https://doi.org/10.4049/jimmunol.0901607

Kotturi, M.F., B. Peters, F. Buendia-Laysa Jr., J. Sidney, C. Oseroff, J. Botten, H. Grey, M.J. Buchmeier, and A. Sette. 2007. The CD8+ T-cell response to lymphocytic choriomeningitis virus involves the L antigen: uncovering new tricks for an old virus. *J. Virol.* 81:4928–4940. https://doi.org/10.1128/JVI.02632-06

Madden, D.R., D.N. Garboczi, and D.C. Wiley. 1993. The antigenic identity of peptide-MHC complexes: a comparison of the conformations of five viral peptides presented by HLA-A2. *Cell.* 75:693–708. https://doi.org/10.1016/0092-8674(93)90490-H

Nielsen, M., C. Lundegaard, P. Worning, S.L. Lauemøller, K. Lamberth, S. Buus, S. Brunak, and O. Lund. 2003. Reliable prediction of T-cell epitopes using neural networks with novel sequence representations. *Protein Sci.* 12:1007–1017. https://doi.org/10.1110/ps.0239403

Vitiello, A., L. Yuan, R.W. Chesnut, J. Sidney, S. Southwood, P. Farness, M.R. Jackson, P.A. Peterson, and A. Sette. 1996. Immunodominance analysis of CTL responses to influenza PR8 virus reveals two new dominant and subdominant Kb-restricted epitopes. *J. Immunol.* 157:5555–5562.
